# Direct Observation of the Myosin Va Recovery Stroke That Contributes to Unidirectional Stepping along Actin

**DOI:** 10.1371/journal.pbio.1001031

**Published:** 2011-04-12

**Authors:** Katsuyuki Shiroguchi, Harvey F. Chin, Diane E. Hannemann, Eiro Muneyuki, Enrique M. De La Cruz, Kazuhiko Kinosita

**Affiliations:** 1 Department of Physics, Faculty of Science and Engineering, Waseda University, Tokyo, Japan; 2 Department of Molecular Biophysics and Biochemistry, Yale University, New Haven, Connecticut, United States of America; 3 Department of Physics, Faculty of Science and Technology, Chuo University, Tokyo, Japan; Stanford University, United States of America

## Abstract

Myosins are ATP-driven linear molecular motors that work as cellular force generators, transporters, and force sensors. These functions are driven by large-scale nucleotide-dependent conformational changes, termed “strokes”; the “power stroke” is the force-generating swinging of the myosin light chain–binding “neck” domain relative to the motor domain “head” while bound to actin; the “recovery stroke” is the necessary initial motion that primes, or “cocks,” myosin while detached from actin. Myosin Va is a processive dimer that steps unidirectionally along actin following a “hand over hand” mechanism in which the trailing head detaches and steps forward ∼72 nm. Despite large rotational Brownian motion of the detached head about a free joint adjoining the two necks, unidirectional stepping is achieved, in part by the power stroke of the attached head that moves the joint forward. However, the power stroke alone cannot fully account for preferential forward site binding since the orientation and angle stability of the detached head, which is determined by the properties of the recovery stroke, dictate actin binding site accessibility. Here, we directly observe the recovery stroke dynamics and fluctuations of myosin Va using a novel, transient caged ATP-controlling system that maintains constant ATP levels through stepwise UV-pulse sequences of varying intensity. We immobilized the neck of monomeric myosin Va on a surface and observed real time motions of bead(s) attached site-specifically to the head. ATP induces a transient swing of the neck to the post-recovery stroke conformation, where it remains for ∼40 s, until ATP hydrolysis products are released. Angle distributions indicate that the post-recovery stroke conformation is stabilized by ≥5 *k*_B_ *T* of energy. The high kinetic and energetic stability of the post-recovery stroke conformation favors preferential binding of the detached head to a forward site 72 nm away. Thus, the recovery stroke contributes to unidirectional stepping of myosin Va.

## Author Summary

Myosin Va is a “two-legged” ATP-dependent linear molecular motor that transports cellular organelles by “stepping” along actin filaments in a processive manner analogous to human walking, the two “feet” alternating between forward and backward positions. During stepping, the lifted leg undergoes rotational Brownian movements around a free joint at the leg–leg junction. Although these movements are random, the lifted foot lands preferentially on forward sites and rarely steps backward. This directional bias arises in part from the forward movement of the junction bending the “ankle” of the attached leg. Here, we show that the lifted foot also plays a role in the direction of stepping by controlling the orientation of its actin-binding site (the “sole”), which dictates the accessibility of potential stepping positions. We observed the ATP-dependent foot orientation and its stabilizing on individual myosin Va molecules in real time under an optical microscope; we show that the lifted foot of walking myosin Va is oriented in a “toe-down” conformation so that binding to a forward site on actin is preferred largely over backward or adjacent sites. Thus, the great kinetic and energetic stability of the myosin Va lifted foot conformation contributes to unidirectional stepping along actin filaments.

## Introduction

Myosin is an ATP-driven linear molecular motor that produces force and unidirectional movement along actin filaments. The “swinging lever arm” hypothesis proposes that small nucleotide-dependent movements at the catalytic ATPase active site are amplified by rotation of the myosin “lever arm,” or “neck,” light chain–binding domain that extends from the motor domain, or “head” [1],[2]. In the myosin chemomechanical cycle, the lever arm swing that propels the myosin motor forward along actin is referred to as the “power stroke” and is accepted as a general mechanism for myosin contractility. The “recovery stroke” is the essential motion that primes, or “cocks,” the lever arm in the pre-power stroke position while myosin is detached from actin. These strokes are the basis for the physiological functions of all characterized myosin motors.

**Figure 1:**
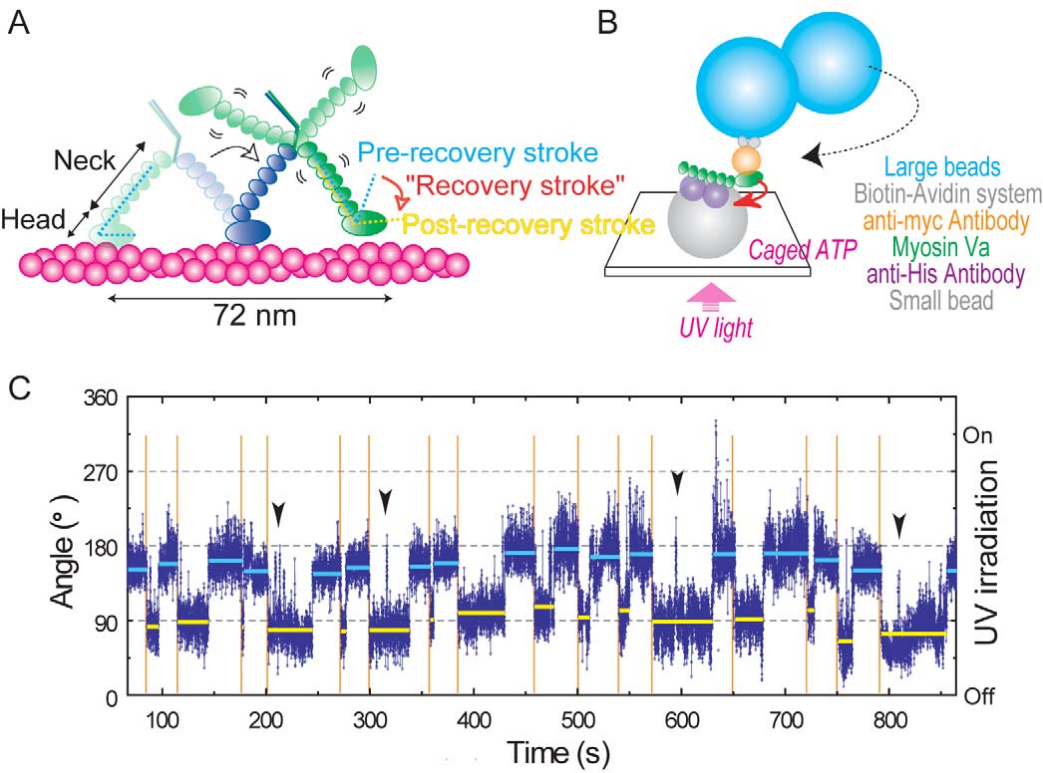
Observation of head–neck swing in monomeric myosin Va in the absence of actin. (A) Postulated recovery stroke during walking of intact myosin Va. Myosin Va has two long necks (blue and green) reinforced with six calmodulin light chains (small ellipsoids) and two catalytic heads (large ellipsoids) that hydrolyze ATP. Walking (toward right) on an actin filament (magenta) begins with binding of ATP to the trailing head to dissociate it from actin. The leading neck (blue) then leans forward, powered by ATP hydrolysis (presumably P*_i_* release) in the leading head. The unbound neck (green) fluctuates around the neck–neck junction until the head binds to a site ∼35 nm ahead of the blue head. If the head–neck angle takes post-recovery stroke conformation, the actin binding surface properly orients to bind actin only when the head is brought forward. (B) Observation system. The neck portion of monomeric (single-headed) myosin Va is fixed on a small bead (diameter, 50 nm; almost invisible) through his-tagged calmodulin(s) and anti-his antibody(s). A streptavidin-coated bead duplex (diameter 290 nm; clearly visible) is attached to the myc-tagged head through biotinylated anti-myc antibody. UV irradiation of caged ATP produces ATP, which is quickly consumed by apyrase in the solution. Nonspecific binding of the neck portion to the small beads occurred as well. (C) A time course of bead swings in response to UV flashes (orange vertical lines) of 0.1-s duration at 100% intensity. Dark blue dots with a gray line, angular positions at 33-ms intervals (video frame rate); cyan, average angles before UV irradiation (pre-recovery stroke state); yellow after irradiation (post-recovery stroke state). Arrow heads, UV uncoupled swings. Dwell time for hypothesized two states are basically determined such that ratio of the two dwell times between each UV irradiations is the highest.

Myosin Va is a cargo transporter in cells [3] that has two heads, each connected to a long and relatively stiff neck [4] reinforced with six calmodulins (Figure 1A). Myosin Va moves processively along actin filament and takes unidirectional “steps” [5] in which it alternately places its two heads in forward positions ∼72 nm away from a previous binding site [6], analogous to human bipedal walking. A mechanism for unidirectional stepping has been investigated and proposed as follows (Figure 1A). When a head is detached off actin, the detached neck undergoes rotational Brownian fluctuations around a free joint at the neck–neck junction [7],[8]. Although the fluctuations are random [7], the power stroke of the bound head [4],[9] tilts the neck via “lever action” and moves the junction (i.e., the pivot point for the fluctuations) forward, thereby favoring binding of the detached head to a forward site.

This mechanism explains how a detached head can access a forward site, but not why it binds preferentially to a forward site 72 nm away as opposed to other accessible sites as, for example, a site adjacent to the bound head. For a detached head to bind actin, the actin-binding site of myosin must be properly oriented with respect to the actin filament. Therefore, since the position of the neck–neck junction relative to the actin filament is constrained by the bound neck, the orientation (angle) and stability of the detached head relative to its neck (head–neck angle) dictate the binding site along a filament. The detached head orientation is determined by the recovery stroke that occurs after ATP-induced detachment from actin. If the role of the recovery stroke were just to prime myosin, the head–neck angle could fluctuate significantly. This could allow for the unbound head to bind to a site near or adjacent to the bound head as well as to a site 72 nm away with similar frequency. Such a distribution of the step size, however, has never been observed in the absence of applied external load [5],[6],[10]. Therefore, another mechanism must exist.

We anticipated that the recovery stroke plays a critical role in orientating the unbound head so that binding to a ∼72-nm forward site occurs preferentially [11],[12]. In addition, it has been reported that myosin Va moves forward under ∼2 pN of backward load [5],[10] which would bring the junction back beyond the neutral position [13] or reverse the power stroke [14], and cancels the bias introduced by the attached head power stroke. The additional role of the recovery stroke above can be another bias for forward stepping even in the presence of the load. Thus, the properties of the recovery stroke are critical for the myosin Va stepping mechanism.

Several recent structural and kinetic studies have demonstrated the existence and implications of the myosin recovery stroke. High-resolution crystal structures of muscle myosin II [15] identified different nucleotide-dependent head–neck angles in the absence of actin; these are thought to correspond to pre- and post-recovery stroke angles. Bulk Förster resonance energy transfer assays of myosin II revealed two [16] or three [17] nucleotide-dependent (averaged) transient angle distributions. In addition, electron microscopic analysis of myosin Va [18] showed two different orientations (i.e., projection angles) of heads relative to the neck, depending on the nucleotide in solution. These observations have contributed to a general model in which ATP binding triggers the recovery stroke, and phosphate (P*_i_*) release after hydrolysis leads to relaxation of the recovery stroke (i.e., generation of the power stroke). However, the energetic and kinetic angle stability of the pre- and post-recovery stroke conformations of myosin (Figure 1A) and the manner in which they contribute to actin binding specificity during processive stepping of myosin Va remains unknown.

We present in this study, to the best of our knowledge, the first direct observations of the myosin recovery stroke (angle change at head–neck junction) in real time and at the single molecule level. We developed a novel light-induced ATP-concentration controlling system and single motor molecule assay that enables the direct observation of the nucleotide-dependent dynamics and fluctuations of the myosin motor domain. Our observations and analysis indicate that the myosin Va motor conformation adopted after the recovery stroke is kinetically and energetically stable, which allows for the detached head to bind preferentially to a forward site 72 nm away, thereby providing the grounds for biased forward stepping of myosin Va along actin filaments.

## Results

### Direct Real-Time Observation of Head–Neck Swing in Myosin Va

We constructed an optical microscope observation system (Figure 1B) to directly visualize in real time the nucleotide-dependent swings (i.e., strokes) and fluctuations of the myosin head–neck angle using an engineered monomeric (single-headed, “S1-like”) myosin Va (Figure S1). We anticipated that a monomeric myosin Va molecule with a 50-nm bead (gray) attached at its neck (configuration depicted in Figure 1B) would permit transient swinging of a 0.29-µm bead duplex (cyan) attached to the distal head region. To determine how the head orientation, assayed from the bead position of the 0.29-µm bead duplex, responds to ATP, we included 200 µM caged ATP and 1.7 mU µl^−1^ apyrase in the solution, such that ultraviolet (UV) irradiation generated an ATP transient that was rapidly removed (hydrolyzed to AMP) by the apyrase with a time constant of 2–3 s (Figures S2 and 2B). We imaged a duplex (or a larger aggregate) of beads, and initiated a full-intensity (∼2 nW µm^−2^, defined as 100%) UV pulse for 0.1 s that yielded a peak ATP concentration ([ATP]_peak_) of ∼2 µM (Figure S2). Approximately 0.1% of duplexes made a distinct angular (>30° judged in real time) swing within several seconds of the UV flash. Such a low frequency is not unexpected given the low probability of an unobstructed configuration, as illustrated in Figure 1B (drawn to scale in Figure S4).

Myosin Va predominantly adopts two distinct conformations during an experiment: a resting angle in the absence of ATP (i.e., before UV irradiation; cyan in Figures 1C, 3A, and S5) and a metastable angle (yellow) accessible only after ATP generation (save rare excursions driven by Brownian fluctuations), interpreted as the post-power stroke and pre-power stroke conformations of myosin Va, respectively.

A large fraction (∼50%) of the beads that swung returned to the original angle in less than 2 min, and the UV-induced transient swings could be repeated multiple (>2) times (Figures 1C and 3A; Video S1). We monitored 15 such duplexes (i.e., myosin Va molecules) and analyzed a total of 121 swing–return pairs as detailed below. A subset (∼20%) made two return swings and then detached from the surface or remained immobile. The remaining ∼30% did not return or did not respond to the second UV flash.

Excursions to the post-recovery stroke “state” (see Figure 1C legend) are ATP (UV flash)–dependent. Every UV irradiation lasting 0.1 s at 100% intensity ([ATP]_peak_ ∼ 2 µM) induced a bead swing within a few seconds (0.78 s on average; 29 flashes in six molecules; e.g., Figure 1C). Shorter and/or weaker irradiations yielded longer delays before a swing (Figure S5A and S5B) or no bead swings. UV irradiation while the bead duplex was in the post-recovery stroke state, in contrast, never induced a swing: 37 flashes of 0.1- or 0.2-s duration at 100% intensity failed to induce bead rotation in six molecules (Figure S5C). Thus, swings from the pre-recovery stroke state are initiated and limited by ATP binding, and myosin Va in the pre-recovery stroke state prior to a swinging event is free of bound nucleotide.

### Quantitative Measurement of ATP Dependence of Head–Neck Swing by Novel ATP-Concentration Controlling System

**Figure 2:**
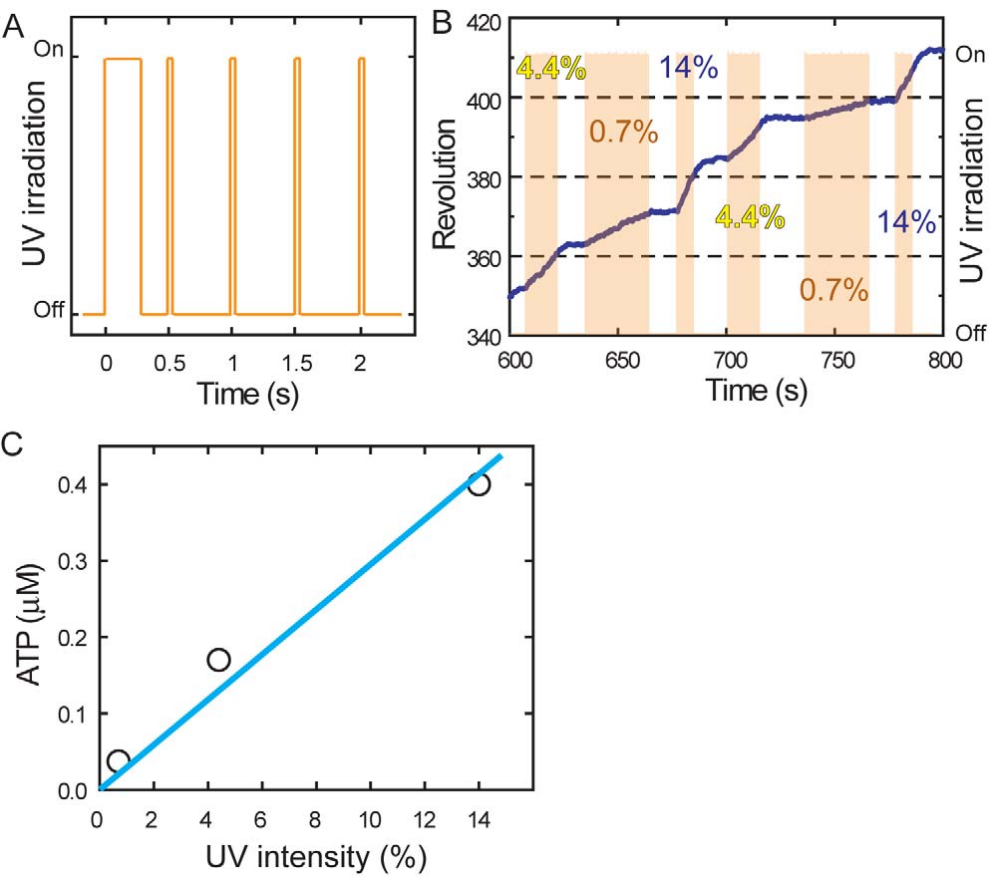
Generation of quasi-stationary [ATP] by patterned UV irradiation and its confirmation by the rotary motor F_1_-ATPase (GT mutant). (A) UV irradiation pattern: one 0.3-s flash followed by 0.05-s flashes at 0.5-s intervals. (B) A rotation time course under indicated UV intensities (orange shading indicates UV flashes). The decelerations after the termination of UV irradiation could be fitted with an exponential as in Figure S2B, with an average decay time for ATP depletion of 3.1±0.5 s, which is similar to that in the case of native myosin Va (Figure S2). (C) Estimated quasi-stationary concentrations of ATP generated under the patterned UV irradiation at indicated intensities. The average rotation speeds in Figure S3B were divided by the slope in Figure S3C. The results are grossly consistent with Figure S2, which indicates the generation of ATP at ∼20 µM s^−1^ at 100% UV: under the irradiation pattern in (A) with the duty ratio of 0.1 (0.05 s/0.5 s), the rate of ATP generation would be ∼2 µM s^−1^, and thus division with the ATP depletion rate of 1/(2–3 s) would predict a steady-state [ATP] at 100% UV of 0.7–1 µM.

To quantitate the ATP dependence of swings, we developed a new technique that generates a nearly constant level of ATP in a chamber using caged ATP, which was first applied to a biological system by Trentham and colleagues [19], and evaluated the method using the rotary F_1_-ATPase (GT mutant) motor [20]–[22] (Figure S3A). Stepwise UV pulse sequences with pulse width modulation (Figure 2A) of varying intensity (14%, 4.4%, and 0.7%) repeatedly generated intensity-dependent rotations of a given F_1_-ATPase molecule (Figure 2B). Averaged traces of rotations are smooth, indicating that the UV pulse sequences generate nearly constant ATP levels in the sub-second time scale (Figure S3B). Rotational rates in the presence of known [ATP] (Figure S3C) yielded UV intensity–dependent ATP concentrations (Figure 2C). These calibrations for constant ATP level allow us to analyze the kinetics of ATP-induced myosin Va swinging.

**Figure 3:**
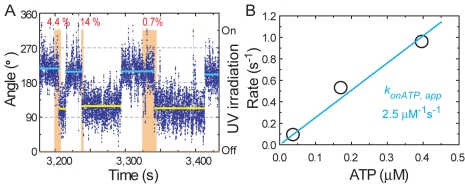
Quantitative measurement of ATP dependence of head–neck swing. (A) A time course of bead swings induced by patterned UV irradiation (orange shading; see Figure 2A) that would produce a nearly constant [ATP] of ∼0.4 µM (at 14% intensity), 0.17 µM (at 4.4%), or 0.037 µM (at 0.7%) (Figure 2C). Color-coded as in Figure 1C. (B) The apparent rate of ATP binding at different concentrations of ATP produced by the patterned UV irradiation. The inverse of the time elapsed before a swing is plotted (averaged over 21, 15, and 28 swings in eight molecules). Apparent rate constant (*k*_onATP, app_) is obtained by linear fitting.

In the myosin Va swing assay, we turned on the sequence at different UV intensities (i.e., [ATP]) until a swing occurred (orange bars in Figures 3A and S5D). The time before a swing was inversely proportional to the [ATP] (Figure 3B), yielding an apparent ATP binding rate constant of 2.5×10^6^ M^−1^s^−1^, comparable to the value of 1.7×10^6^ M^−1^s^−1^ measured in solution (Protocol S1). These qualitative measurements strongly suggest observed swinging events are those of functional myosin motors.

### Kinetic Analysis of Post-Recovery Stroke State

**Figure 4:**
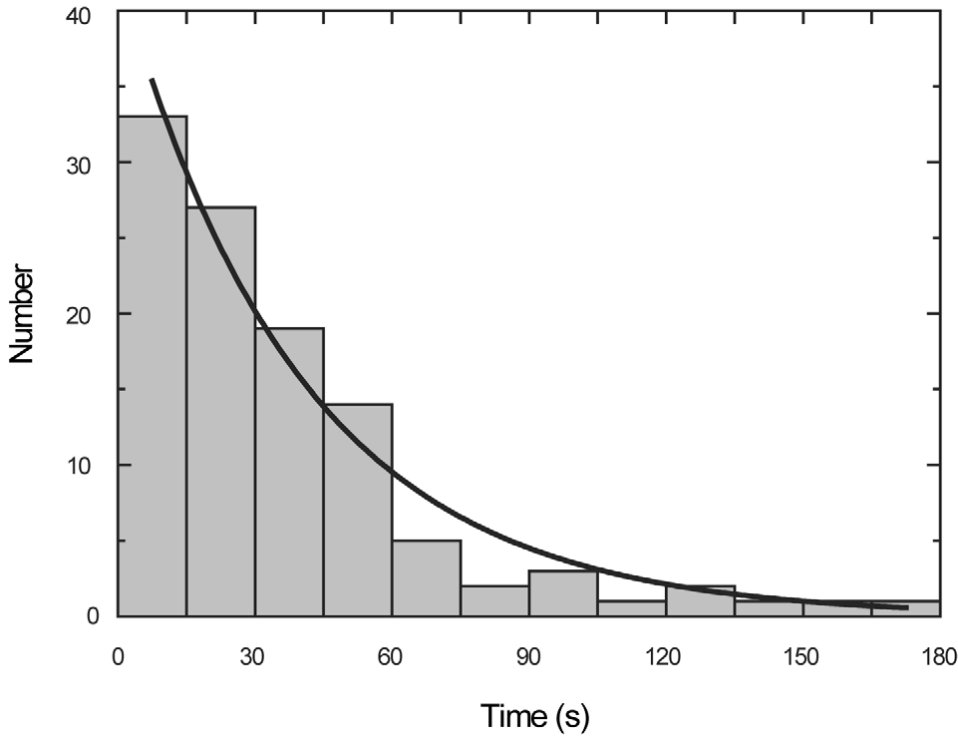
Dwell times in the post-recovery stroke state. The histogram for 109 swings (without an additional UV flash during the post-recovery stroke state) in 15 molecules is fitted with an exponential of the time constant 40±4 s (standard error).

Except for occasional, short reversals in the post-recovery stroke state (e.g., arrow heads in Figures 1C and S6; discussed below), the post-recovery stroke state is characterized by exponentially distributed dwell times with an average of 40±4 s (standard error) (Figure 4). Note that the post-recovery stroke state is quite stable kinetically, particularly in comparison to the stepping intervals of 60–80 ms at physiological [ATP], during which P*_i_* release is accelerated by binding to actin [10],[23]. Our bulk, biochemical assays indicate that ATP is rapidly (>100 s^−1^; [23]) hydrolyzed into ADP and inorganic P*_i_* is released with a rate constant of ∼0.02 s^−1^ (τ = ∼50 s) (Figure S7). Measurements with a shorter-neck, 1IQ construct [23],[24] indicated a P*_i_* release rate constant of ∼0.02 s^−1^ and a subsequent ADP release rate of ∼1.2 s^−1^. Collectively, these measurements indicate that myosin Va in the post-recovery stroke conformation has ADP and P*_i_* bound in its active site and that P*_i_* release limits the return swing (i.e., power stroke off actin), consistent with bulk Förster resonance energy transfer assays with myosin II in solution [16] and electron microscopy of myosin Va bound to actin [25].

### Stability of Conformation in Post-Recovery Stroke State

**Figure 5:**
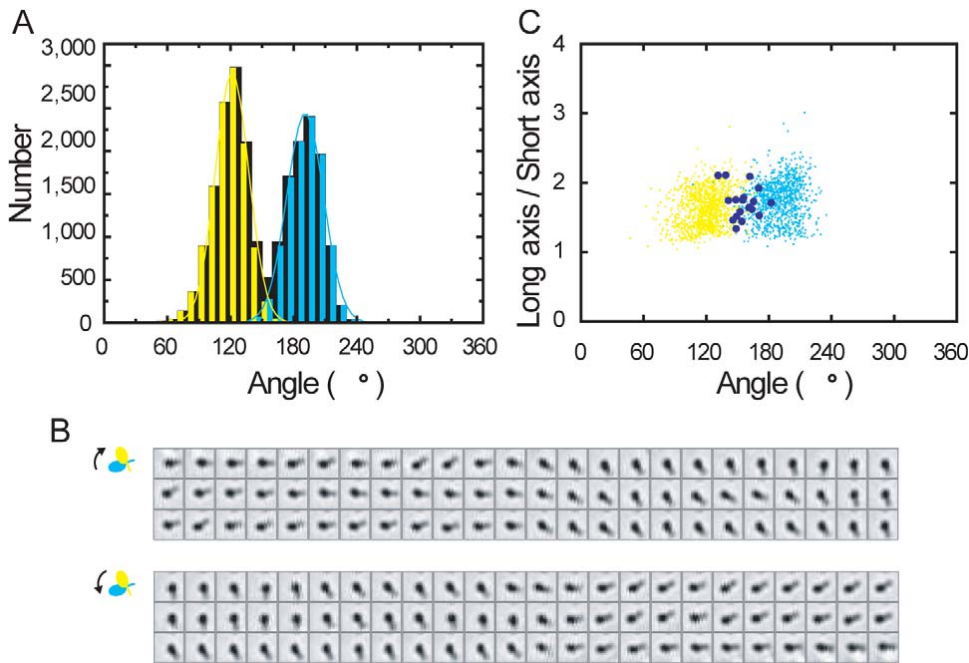
Swing angles. (A) Angle (θ) distribution for a swinging bead aggregate (time course in Figure S5A). Black indicates all frames; cyan indicates pre-recovery stroke state; yellow indicates post-recovery stroke state. Lines show Gaussian fits: exp[−(θ − θ_m_)^2^/2σ^2^] where θ_m_ is the mean angle. (B) Sequential images (1.8-µm square) at 33-ms intervals of the aggregated beads in three swings. The whole time course is analyzed in (A). (C) The axial ratio (long/short axis) of the bead image in (B), analyzed over the whole time course in Figure S5A. Cyan indicates pre-recovery stroke state; yellow indicates post-recovery stroke state; dark blue indicates during swings. Cyan and yellow dots are shown for every ten video frames for clarity.

The angular fluctuations in both the pre-recovery stroke and post-recovery stroke states are well fitted to Gaussian distributions (Figures 5A and S8), with a peak separation yielding an average swing amplitude (θ_swing_) of 85°±19° (standard deviation [s.d.] for 15 molecules). The measured amplitudes reflect projections in the image plane, and thus the actual amplitudes will differ if out-of-plane swinging occurred. However, the observation that the appearance of most (∼2/3) of the bead amplitudes is independent of the swing angle (e.g., Figure 5B), as confirmed by the constancy of the axial ratio (Figure 5C), indicates that the recorded swings used in the analysis were in a near horizontal plane. Electron micrographs of myosin Va without actin show comparable (∼90°) nucleotide-dependent angular changes [18], thereby strengthening the interpretation that transitions between pre-recovery stroke and post-recovery stroke conformations of myosin Va are being observed.

Both the pre-recovery and post-recovery stroke conformations display considerable conformational flexibility, as indicated by the standard deviation of the angular fluctuations (Figures 5A and S8). The magnitudes of fluctuations in both states are comparable, with the Gaussian width (s.d., σ) averaging 24°±10° (s.d. for 15 molecules; *σ*_pre_/θ_swing_ = 0.29±0.09) for the pre-recovery stroke conformation and 26°±9° (*σ*_post_/θ_swing_ = 0.31±0.10) for the post-recovery stroke conformation. These observed fluctuations include contributions from flexibility in the myosin–bead junctions as well as experimental image noise, so they represent an upper limit, with actual angle fluctuations being smaller.

The Gaussian width of the thermally driven fluctuations (σ) measured here, with the equipartition principle [26],
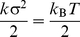
(1)where *k*_B_ *T* (4.1 pN•nm) is thermal energy and *k* is the myosin Va head–neck joint stiffness (spring constant), allows us to determine the spring constants, *k*_pre_ = 23 pN•nm•rad^−2^, and *k*_post_ = 20 pN•nm•rad^−2^_._

With this spring constant, the energy required for bending of the head in the post-recovery stroke conformation to the pre-recovery stroke conformation (i.e., the energy needed to bend the spring by θ_swing_) expressed in terms of the elastic potential energy (*E*),
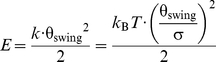
(2)is 5.2 *k*_B_ *T*. The post-recovery stroke conformation is stabilized at least to this extent: because the experimental σ in equation 1 includes the fluctuations of other components described above, the spring constant *k* for the head–neck junction must be underestimated, and thus the energy difference, *E*, of 5.2 *k*_B_ *T* between pre- and post-recovery stroke conformations is a lower limit.

There were occasions where we observed momentary swings back to the pre-recovery stroke angle in the post-recovery stroke state (e.g., arrow heads in Figures 1C and S6). These are unlikely to be purely Brownian excursions, because the bead tended to remain at the pre-recovery stroke angle for a second or longer. A natural return followed by immediate ATP binding that would induce a second swing is also unlikely, because [ATP] must be negligibly low and these momentary swings happened irrespective of the time after UV irradiation. The observed momentary returns may represent reversal of the reaction responsible for the swing to post-recovery stroke conformation, ATP hydrolysis [23], or subsequent myosin isomerization [16]. We note that the return frequency in the absence of drag from the attached beads could possibly be higher.

## Discussion

### A Role of the Recovery Stroke in Myosin Va Unidirectional Stepping

**Figure 6:**
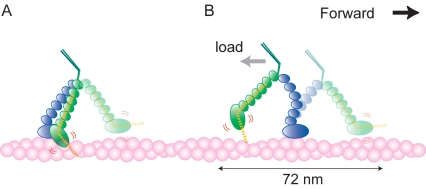
A role of angle stability after recovery stroke for myosin Va unidirectional stepping. Light-colored myosin Va makes forward stepping as in Figure 1A. Yellow dotted lines indicate the head–neck angle of the post-recovery stroke conformation. (A) Stability of the head–neck angle in the post-recovery stroke conformation prevents the unbound head from binding to an adjacent site of the bound head. (B) Stability of the head–neck angle in the post-recovery stroke conformation prevents the unbound head from binding to a backward site even in the presence of backward load.

The natural assumption is that the detached head accessing a forward site in the post-recovery stroke conformation will have its actin binding site properly oriented for productive binding to actin (Figure 1A). Conversely, when the detached head goes back to the post-recovery stroke conformation, the actin binding site is predicted to be oriented incorrectly, thereby precluding actin binding (Figure 6A). The kinetic stability of the post-recovery stroke state observed here indicates that this proper head orientation is maintained for ∼40 s, much longer than the stepping intervals. Even if the head in the post-recovery stroke state accidentally touches a backward site at a moment when the head adopts a near pre-recovery angle by fluctuation or momentary reversal, the binding should be unstable by at least by 5 *k*_B_ *T* compared to forward binding. Thus, the kinetic and energetic stabilities of the post-recovery stroke state together ensure forward binding of an unbound head.

Momentary binding of a head with incorrect orientation will be unstable from intramolecular strain [11],[12]. Consistently, a quantitative model has shown that the lever arm (neck) elasticity and its strain influence the position of the next binding site on actin, therefore the detached head preferentially binds to the forward site [27]. This model assumes that the unbound neck with bound ADP–P*_i_* rigidly takes post-recovery stroke conformation, which we report here.

The key for directional movement is to bias the completely random Brownian rotations of a detached neck toward forward binding. The power stroke and its angle stability of the attached rear head contribute approximately half of the bias by moving the pivot for the Brownian rotation of the unbound neck forward, which allows the detached head to access positions 36- to 72–nm distant on an actin filament [4],[7] (Figure 1A). The remaining bias between positions ∼36 nm and ∼72 nm from the detached site is provided by the recovery stroke and its stability. Under a high backward external load, the power stroke would fail to produce a bias: owing to the compliance in the neck and/or neck–head junction [13],[14], the neck–neck junction would be pulled back to the neutral position, immediately above the bound head (Figure 6B). Even under this circumstance, the bias by recovery stroke still works, favoring forward binding. Therefore, for ∼72-nm discrete unidirectional steps of myosin Va, the recovery stroke and its angle stability of the detached head contributes to the bias, in addition to the power stroke and its angle stability of the attached head. This mechanism may contribute to transport cargos in a cell since some cellular components could be obstacles to hinder the movement of cargo at times.

An alternative mechanism has recently been proposed for myosin VI [28], which is thought to function as a force sensor as well as a transporter [29]: stable lead head binding is facilitated by a backward load on the head, and hence internal strain between the two necks promotes forward binding of an unbound head. Myosin VI is the only reverse motor known to date, moving in the direction opposite to all other myosins studied so far. It is of interest to study whether the other myosins, including myosin Va, also adopt a similar, strain-dependent binding for forward bias.

The stability of the post-recovery stroke conformation would also be important for muscle myosin II, which can produce tension without contraction (isometric tension) by repeatedly “scratching” actin. Forward binding is required for efficient force production, but the base of the necks does not move in this situation, and thus myosin II may rely entirely on the head orientation being stabilized in the post-recovery stroke state. Other linear motors may also rely on an effective swing to the post-recovery stroke conformation [11],[12].

### Microscopic Observations with Constant and Changeable ATP Level for a Variable Time

To study ligand-dependent motion of molecules, caged nucleotides (uncaged by UV irradiation) have been combined with microscopic observations. UV pulse irradiation allows one to trigger motion of the molecular motor and to clearly show its nucleotide dependence [30],[31], and modulation of UV irradiation time allows one to control motor velocity and total movement [32]. This assay design has the advantage over conventional flow/mixing assays in that solution conditions (e.g., nucleotide concentration) can be altered rapidly and with minimal perturbation. However, caged nucleotide measurements have been limited to kinetic analysis because the concentration of uncaged compound can change significantly during the course of an experiment, particularly if consumed by the system being examined (i.e., diffusion, enzyme–substrate interaction, or apyrase). We have developed a new technique to keep ATP level constant in which the concentration and time evolution can be modulated by light intensity and irradiation time (Figures 2, 3A, S3, and S5D). This method for visualization of a nucleotide-linked conformational change in a motor protein under the controlled delivery of ATP should be generally applicable to ligand-induced conformational changes of macromolecules.

## Materials and Methods

### Materials

Monomeric *Gallus gallus* myosin Va truncated at Leu-909 (containing all six IQ motifs) with an N-terminal myc tag (EQKLISEEDL) positioned directly replacing Met-1 and a C-terminal FLAG tag (DYKDDDDK) with a single glycine linker (Figure S1) was co-expressed with Lc-1sa in Sf9 cells and purified by FLAG affinity chromatography in the presence of excess calmodulin as previously described [24],[33]. The calmodulins on the expressed protein were exchanged for 6× his-tagged calmodulin, expressed in *Escherichia coli*, as previously reported [34] and modified [7]: the his-tagged calmodulin and monomeric myosin Va at the molar ratio of 6∶1 were mixed and incubated for 10 min on ice in 20 mM imidazole-HCl (pH 7.6), 4 mM MgCl_2_, 100 mM KCl, 0.04 mM EGTA, 0.5% (v/v) β-mercaptoethanol, and 400 µM CaCl_2_. The reaction was terminated by the addition of 4 mM EGTA followed by >20 min incubation on ice. Monomeric myosin Va carrying his-tagged calmodulin was mixed with an anti-his monoclonal antibody (Clontech Laboratories) at the antibody:myosin molar ratio of 17∶1 in buffer A (25 mM imidazole-HCl [pH 7.6], 4 mM MgCl_2_, 100 mM KCl, 1 mM EGTA, 5 mM DTT), and incubated at room temperature for >5 min to allow binding.

### Swing Assay

A flow chamber, in all experiments under a microscope, was made of two coverslips separated by two spacers of ∼100-µm thickness, and, after the last infusion, the chamber was sealed with silicone grease or nail liquid. The following infusions (2–3 chamber volumes), all in buffer A, were made with 1–2 min of incubation in between: 2 mg ml^−1^ unphosphorylated α-casein for surface blocking, buffer A for washing, 5.6% (w/v) 0.05-µm silica beads (Polysciences), buffer A for washing, monomeric myosin Va (10 nM) complexed with anti-his antibody (for binding to the silica beads through the antibody) or myosin Va alone without the calmodulin exchange (for direct binding), 2 mg ml^−1^ unphosphorylated α-casein, 25 µg ml^−1^ biotinylated anti-myc monoclonal antibody (Millipore), and buffer A for washing. Finally, 0.29-µm streptavidin-coated beads (Seradyn), washed three times by centrifugation in buffer A, were infused together with 200 µM caged ATP (Dojindo), 1.7 mU µl^−1^ apyrase (Sigma), 1.1 mg ml^−1^ unphosphorylated α-casein, and 0.5% (v/v) β-mercaptoethanol.

The purpose of the anti-his antibody was to let it serve as a cushion between the myosin neck and a silica bead so as to keep the myosin intact. Direct binding, though, worked as well, and some results, e.g., in Figures 1C and S5A–S5C, were obtained with direct binding. In both cases, most of the 0.29-µm beads on the surface were bound to the head of myosin Va through a biotin–avidin linkage, because the bead density decreased significantly without myosin, with non-biotinylated anti-myc antibody instead of the biotinylated one, or by mixing excess biotin with the streptavidin-coated beads before infusion. When we infused short actin filaments instead of the 0.29-µm beads, they attached (presumably) to myosin Va, and a flash of 100% UV light for 0.2 s released >97% of them from the surface within a few seconds.

### Microscopy

We used an Olympus IX70 microscope equipped with a 100× objective (UPLSAPO100× O IR, N.A. 1.4, Olympus), a stable sample stage (KS-O, ChuukoushaSeisakujo), a dual-view system [35] for simultaneous observation of fluorescence and bright-field images [36], a regular epi-fluorescence port, and an additional UV excitation port consisting of a mercury lamp, an extension tube (IX2N-FL-1) that forms an intermediate (conjugate) image plane outside the microscope body, and a computer-controlled shutter with 5-ms open–close time (Uniblitz). Fluorescence of Alexa 488 was excited at 475–490 nm, and images at 500–535 nm were captured with an intensified (VS4-1845, Video Scope) CCD camera (CCD-300-RCX, Dage-MTI). Bright-field images (650–730 nm) were recorded with another CCD camera. UV excitation (300–400 nm) for uncaging ATP was confined in a circle of diameter ∼90 µm at the image plane. A mask was placed on the conjugate plane in the extension tube such that the central ∼30-µm square in the image plane did not receive UV light. The swing assay was always made near the center of the masked area to protect myosin from possible UV damage, although we found that direct UV irradiation at the maximum intensity (see below) for tens of seconds did not affect the motile activity of myosin Va. The rotation speed of F_1_-ATPase (for estimation of ATP concentration; Figures 2 and S3) did not depend on the position in, and even outside, the masked area, and short actin filaments bound to myosin Va were released by a UV flash with indistinguishable kinetics at all positions. Note that oblique UV beams illuminated the solution above the masked area except for the immediate vicinity of the coverslip surface. To record correlation of events and UV irradiation, a part of the UV beam was recorded with the intensified CCD camera above, or with the camera for bright field at an edge of the image. The UV power was measured above the objective lens, and the estimated intensity in the image plane was ∼2 nW µm^−2^ for unattenuated (maximal) excitation (defined as 100% intensity). Observations were made at 23 °C.

### Image Analyses

The orientation of a bead duplex was determined as previously reported [7]. When another bead came nearby, the orientation was judged by eye or abandoned. Ellipticity of a bead image was estimated as the ratio of the long axis length to the short one, calculated from the second moments of the intensity distribution as <*Ix* ^2^>^1/2^/<*Iy* ^2^>^1/2^ where *x* and *y* are pixel coordinates measured along the long and short axes and with the origin at the image centroid, *I* is the pixel intensity minus a threshold value, and <> denotes averaging.

### Estimation of UV-Generated ATP Concentrations

UV-generated ATP concentrations were estimated by both gliding bead assay for native myosin Va and rotational assay for F_1_-ATPase (Protocol S1).

### Bulk Transient Kinetics Assays

ATP binding rate and P*_i_* release rate of myosin Va were measured using stopped flow apparatus (Protocol S1).

## Supporting Information

Figure S1 **Myosin Va construct used in this study.** Amino acid residues are shown by single letters. Sequence numbers in parentheses refer to the original full-length construct. Note that the second amino acid (Ala) in chicken myosin Va is seen in a crystal structure [15], suggesting that the N-terminus takes a stable conformation. Moreover, though only pre-recovery stroke conformation has been solved by high resolution for myosin Va [15], for myosin II, the N-terminal domain consists of a head (motor domain) that takes distinct angle (∼70°) relative to the neck portion (lever arm) in pre-recovery stroke and post-recovery stroke conformations [15].
(JPG)

Figure S2 **Estimation of UV-generated ATP concentration and its decay time by the gliding bead assay.** (A) Time courses of the gliding of a myosin-coated bead on actin after a 100% UV flash for 0.1 s (indicated in orange). Those beads that moved straight (because the actin filament was straight on a surface) were selected for the analysis. Different colors show different beads, dark blue being the average of all records. (B) Displacement records averaged over five or more moving beads, as in (A) (dark blue), in five different chambers distinguished by color. The UV intensity was 100% and duration, 0.1 s. The time courses were fitted with an exponential (smooth lines), giving an average time constant of 1.8±0.3 s (s.d. for the five records shown) for the decay of [ATP] by apyrase. (C) The initial ATP concentration generated by a single UV flash of varying duration at 100% UV intensity. The initial gliding velocity estimated from the exponential fit as in (B) was converted to [ATP] by assuming that the native myosin Va carrying the bead made 36-nm steps by binding ATP at the rate constant of 0.9×10^6^ M^−1^s^−1^ [23]. A linear fit (broken line) indicates that, at 100% UV intensity, ATP is generated at a rate of ∼20 µM s^−1^. A separate set of experiments (not shown) indicated that this rate is proportional to the UV intensity between 0.7%–100%. Bars, standard error.
(JPG)

Figure S3 **Generation of quasi-stationary [ATP] confirmed by the rotary motor F_1_-ATPase (GT mutant).** (A) Observation system (not to scale). The stator (gray; α_3_β_3_ subunit) is adsorbed on a glass surface, and a duplex of streptavidin-coated beads is attached to the biotinylated rotor (black; γ subunit). (B) Rotation of three molecules (a–c) under different UV intensities (color-coded as in Figure 2B). Each molecule was subjected to different intensities repeatedly as in Figure 2B, which is a partial record for molecule b, and each curve in (B) represents an average of >6 rotation time courses obtained under the same intensity. (C) ATP dependence of the rotational speed with regular ATP. Bead duplexes that rotated relatively fast and smoothly were selected, and the average speed over >20 contiguous revolutions (ten for one molecule at 0.05 µM ATP) was determined. The apparent rate constant of ATP binding, based on the assumed consumption of three ATP molecules per turn, is 8.1 ( =  2.7 × 3) µM^−1^s^−1^, comparable with the values previously reported for this mutant (1.8 µM^−1^s^−1^ in a rotation assay, 4.2 or 6.8 µM^−1^s^−1^ for bulk ATPase activity) [21],[22].
(JPG)

Figure S4 **Configuration of myosin and beads drawn to scale.** Examples of configurations in which large duplex beads can swing (A) and cannot swing (B). Myosin (in green) is between two sizes of beads: other proteins shown in Figure 1B are not shown here. The myosin neck is immobilized on a small gray bead, and the head is attached to a large blue duplex. Myosin binding to small and large beads occurs by chance. Duplex bead swinging occurs only when conditions under which the swinging beads do not collide with the surface are satisfied: (i) myosin is on the top of the small bead, (ii) myosin is properly oriented such that the swing plane is parallel to the surface, and (iii) the long axis of duplex beads is almost parallel to a surface. These conditions contribute to a low frequency of observed bead swinging.
(JPG)

Figure S5 **Head–neck swings of myosin Va under different UV irradiation conditions.** Dark blue dots with a light gray line indicate angular positions of the beads on the head at 33-ms intervals (video frame rate); horizontal cyan lines indicate average angles before UV irradiation (pre-recovery stroke state); yellow lines indicate average angles after irradiation (post-recovery stroke state). (A) Single UV flashes of different powers. Vertical cyan lines indicate 100% intensity for 10 ms; yellow indicates 25% for 10 ms; brown indicates 25% for 100 ms. Compared to Figure 1C, where a 100-ms flash at 100% intensity always induced a return swing, short (10 ms) and/or weak (25%) flashes here often had to be applied several times before a successful return swing was observed, indicating that the swings depend on UV-generated ATP. (B) Continuous UV irradiation at 100% and 0.7% intensities for 2 s and 10 s, respectively. Under 100% UV, a swing was observed at 0.74 s on average (seven swings in three molecules), and under 0.7%, at 3.2 s (eight swings). Under continuous irradiation, [ATP] would rise toward the steady-state value of ∼50 µM at 100% (the generation rate of ∼20 µM divided by the depletion rate of 1/[2–3 s]) or ∼0.4 µM at 0.7%, with the time constant of 2–3 s (Figures S2 and S3). The observed waiting times above are thus consistent with ATP binding to myosin Va with the bimolecular rate constant of 1.7×10^6^ M^−1^s^−1^ measured in the stopped flow apparatus (Protocol S1). (C) UV flashes (100%, 0.2 s) in the post-recovery stroke state. None induced a swing back to the pre-recovery stroke state. (D) Quasi-steady ATP levels generated by the patterned irradiation in Figure 2A at indicated intensities. This is another example of the experiment in Figure 3A.
(JPG)

Figure S6 **Momentary reversals to the pre-recovery stroke angle during post-recovery stroke states.** (A and B) Dark blue dots with a light gray line indicate the angular positions of the beads on the head at 33-ms intervals (video frame rate); yellow indicates the average angle of the post-recovery stroke state; cyan indicates the pre-recovery stroke state before (left) and after (right) the shown post-recovery stroke state. These are expanded parts of the time course in Figure 1C, around two arrow heads.
(JPG)

Figure S7 **Time course of fluorescence change after mixing 1.0 µM monomeric myosin Va with 0.4 µM Mg-mantATP.** The increase in fluorescence represents mantATP binding. The reduction results from mantADP release, which is limited by P*_i_* release [23]. The data (gray) represent an individual, unaveraged time course of fluorescence change after subtraction of a baseline from mantATP photobleaching. The smooth line (cyan) through the data represents the best fit and yields a mantATP association rate constant of 1.57 (± 0.002) µM^−1^s^−1^ and a P*_i_* release rate constant of 0.019 (± 0.001) s^−1^. A P*_i_* release rate constant measured with ATP was 0.028 (± 0.001) s^−1^. These are consistent with our previous measurements for a shorter neck construct (P*_i_* release, 0.02 s^−1^) [23].
(JPG)

Figure S8 **Distributions of bead angles in the pre-recovery stroke (cyan) and post-recovery stroke (yellow) states.** Black bars indicate whole frames. These are additional examples of the analysis in Figure 5A. (A–E) Distributions for Figures 1C, 3A, and S5B–S5D, respectively. Lines show Gaussian fits: exp[−(θ − θ_m_)^2^/2σ^2^] where θ_m_ is the mean angle.
(JPG)

Protocol S1 **Materials, gliding bead assay, F_1_-ATPase rotation assay, and transient kinetic analysis.** The detailed protocols are described.
(DOC)

Video S1 **Motion of an aggregate of beads (0.29 µm in diameter) attached to the head of monomeric myosin Va.** Contrast and brightness have been modified (30 frames s^−1^). White bars that appear in the left panel indicate UV irradiations. The video presents approximately the interval 260 s to 370 s in the time course of Figure 1C.
(AVI)

## Abbreviations

P*_i_*: phosphate
s.d.: standard deviation
UV: ultraviolet
