# Supplementary figures and images for: Direct Observation of the Myosin Va Recovery Stroke That Contributes to Unidirectional Stepping along Actin
Source: PLoS Biol. 2011 Apr 12;9(4):e1001031. doi: 10.1371/journal.pbio.1001031 (PMC3075224; doi:10.1371/journal.pbio.1001031)

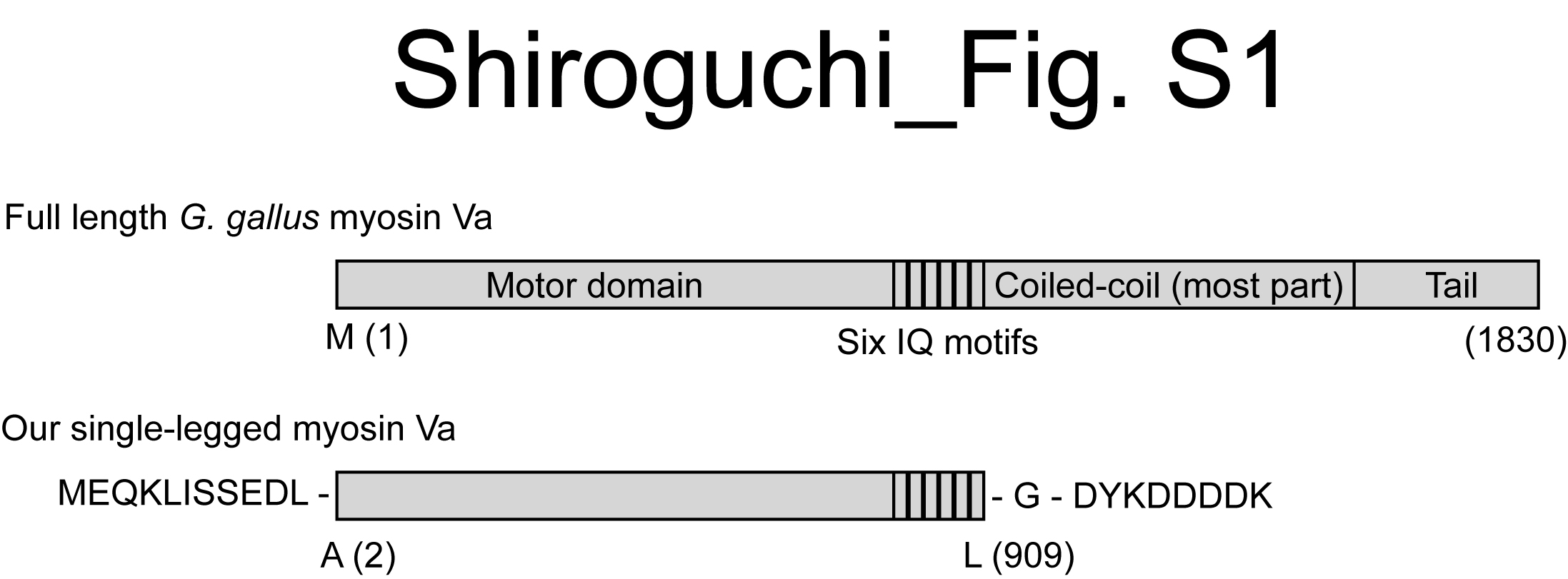

Supplement: Figure S1 — Myosin Va construct used in this study. Amino acid residues are shown by single letters. Sequence numbers in parentheses refer to the original full-length construct. Note that the second amino acid (Ala) in chicken myosin Va is seen in a crystal structure [15], suggesting that the N-terminus takes a stable conformation. Moreover, though only pre-recovery stroke conformation has been solved by high resolution for myosin Va [15], for myosin II, the N-terminal domain consists of a head (motor domain) that takes distinct angle (∼70°) relative to the neck portion (lever arm) in pre-recovery stroke and post-recovery stroke conformations [15]. (JPG) [file pbio.1001031.s001.jpg]

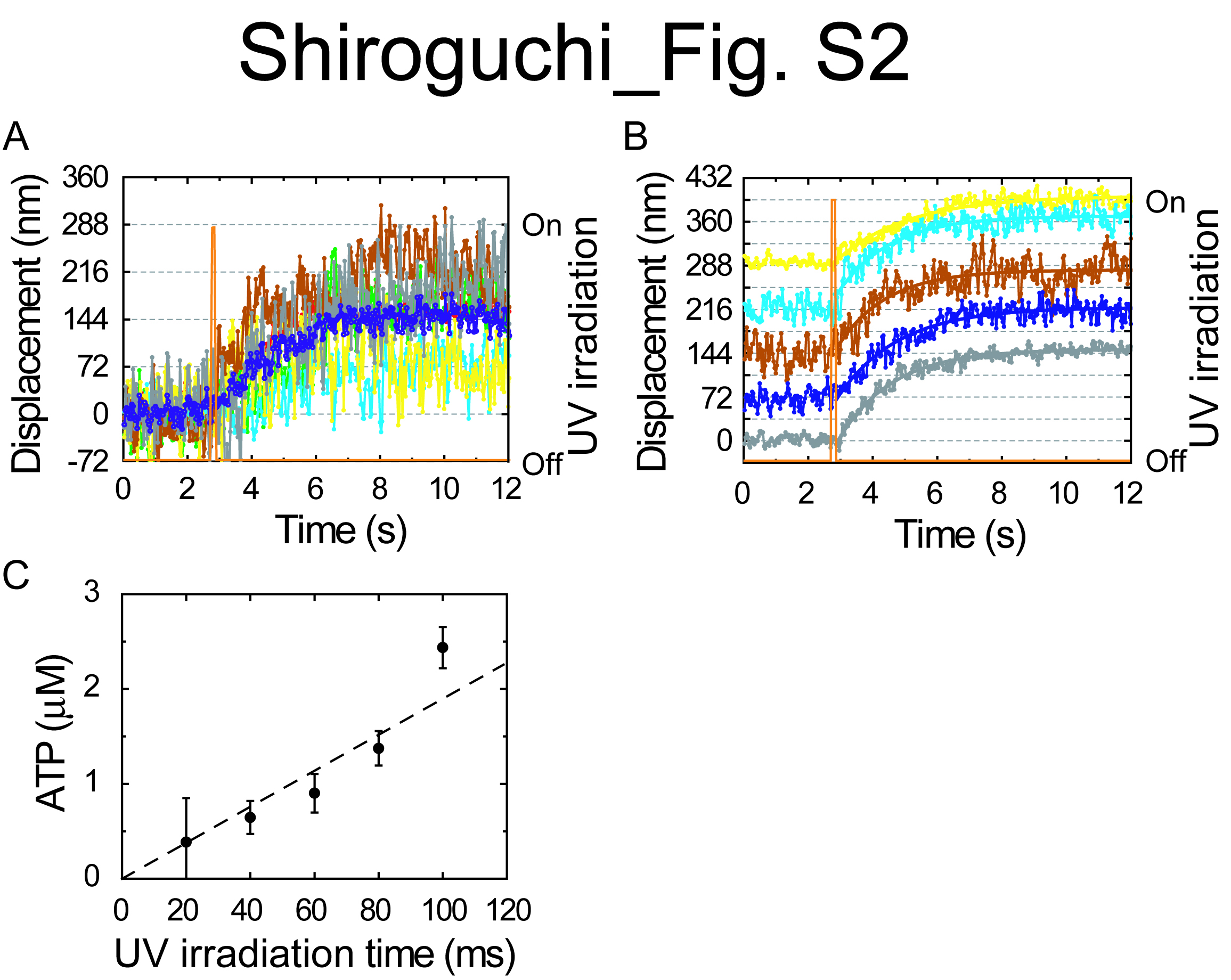

Supplement: Figure S2 — Estimation of UV-generated ATP concentration and its decay time by the gliding bead assay. (A) Time courses of the gliding of a myosin-coated bead on actin after a 100% UV flash for 0.1 s (indicated in orange). Those beads that moved straight (because the actin filament was straight on a surface) were selected for the analysis. Different colors show different beads, dark blue being the average of all records. (B) Displacement records averaged over five or more moving beads, as in (A) (dark blue), in five different chambers distinguished by color. The UV intensity was 100% and duration, 0.1 s. The time courses were fitted with an exponential (smooth lines), giving an average time constant of 1.8±0.3 s (s.d. for the five records shown) for the decay of [ATP] by apyrase. (C) The initial ATP concentration generated by a single UV flash of varying duration at 100% UV intensity. The initial gliding velocity estimated from the exponential fit as in (B) was converted to [ATP] by assuming that the native myosin Va carrying the bead made 36-nm steps by binding ATP at the rate constant of 0.9×10^6^ M−1s−1 [23]. A linear fit (broken line) indicates that, at 100% UV intensity, ATP is generated at a rate of ∼20 µM s−1. A separate set of experiments (not shown) indicated that this rate is proportional to the UV intensity between 0.7%–100%. Bars, standard error. (JPG) [file pbio.1001031.s002.jpg]

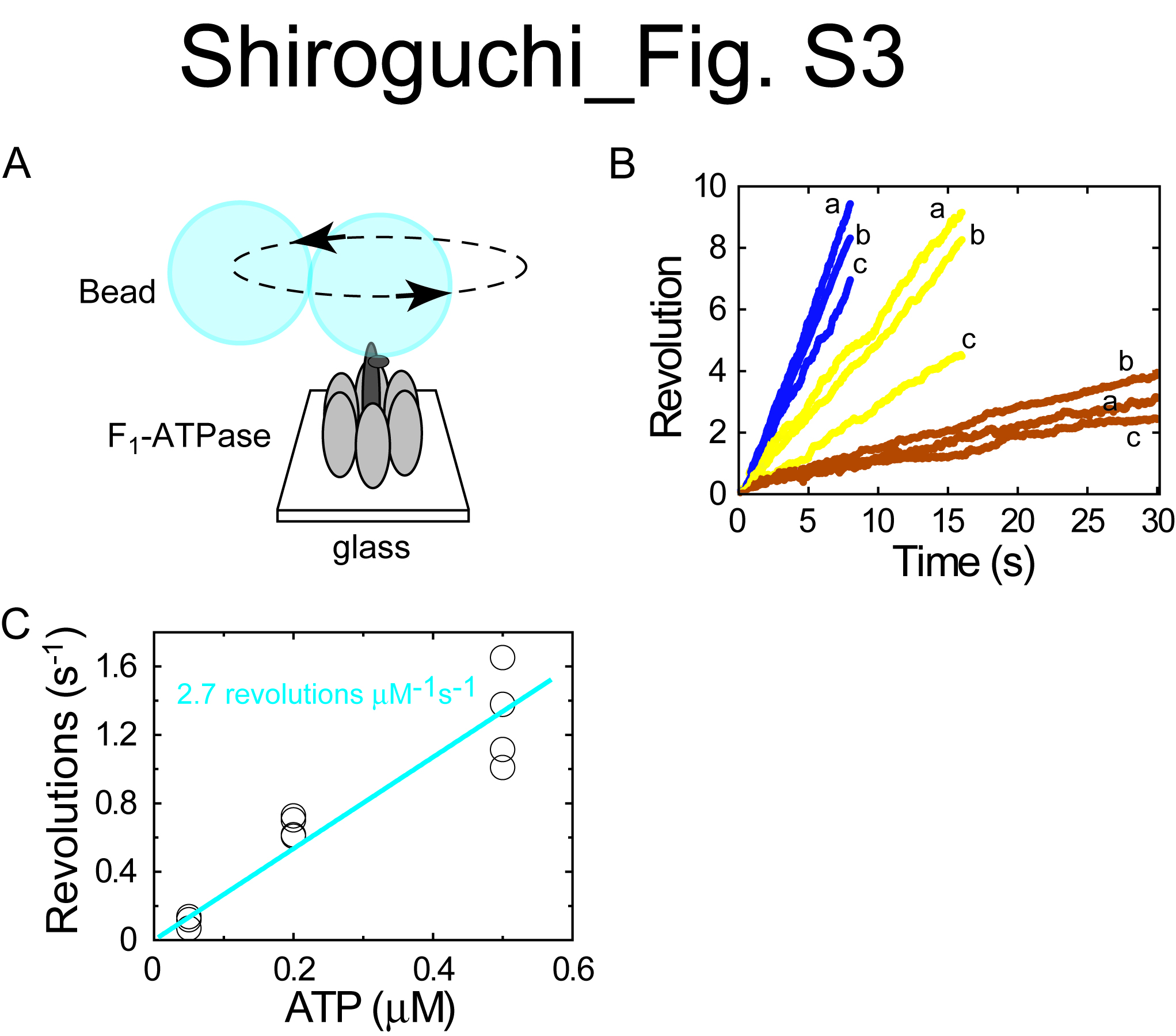

Supplement: Figure S3 — Generation of quasi-stationary [ATP] confirmed by the rotary motor F1-ATPase (GT mutant). (A) Observation system (not to scale). The stator (gray; α3β3 subunit) is adsorbed on a glass surface, and a duplex of streptavidin-coated beads is attached to the biotinylated rotor (black; γ subunit). (B) Rotation of three molecules (a–c) under different UV intensities (color-coded as in Figure 2B). Each molecule was subjected to different intensities repeatedly as in Figure 2B, which is a partial record for molecule b, and each curve in (B) represents an average of >6 rotation time courses obtained under the same intensity. (C) ATP dependence of the rotational speed with regular ATP. Bead duplexes that rotated relatively fast and smoothly were selected, and the average speed over >20 contiguous revolutions (ten for one molecule at 0.05 µM ATP) was determined. The apparent rate constant of ATP binding, based on the assumed consumption of three ATP molecules per turn, is 8.1 ( = 2.7 × 3) µM−1s−1, comparable with the values previously reported for this mutant (1.8 µM−1s−1 in a rotation assay, 4.2 or 6.8 µM−1s−1 for bulk ATPase activity) [21],[22]. (JPG) [file pbio.1001031.s003.jpg]

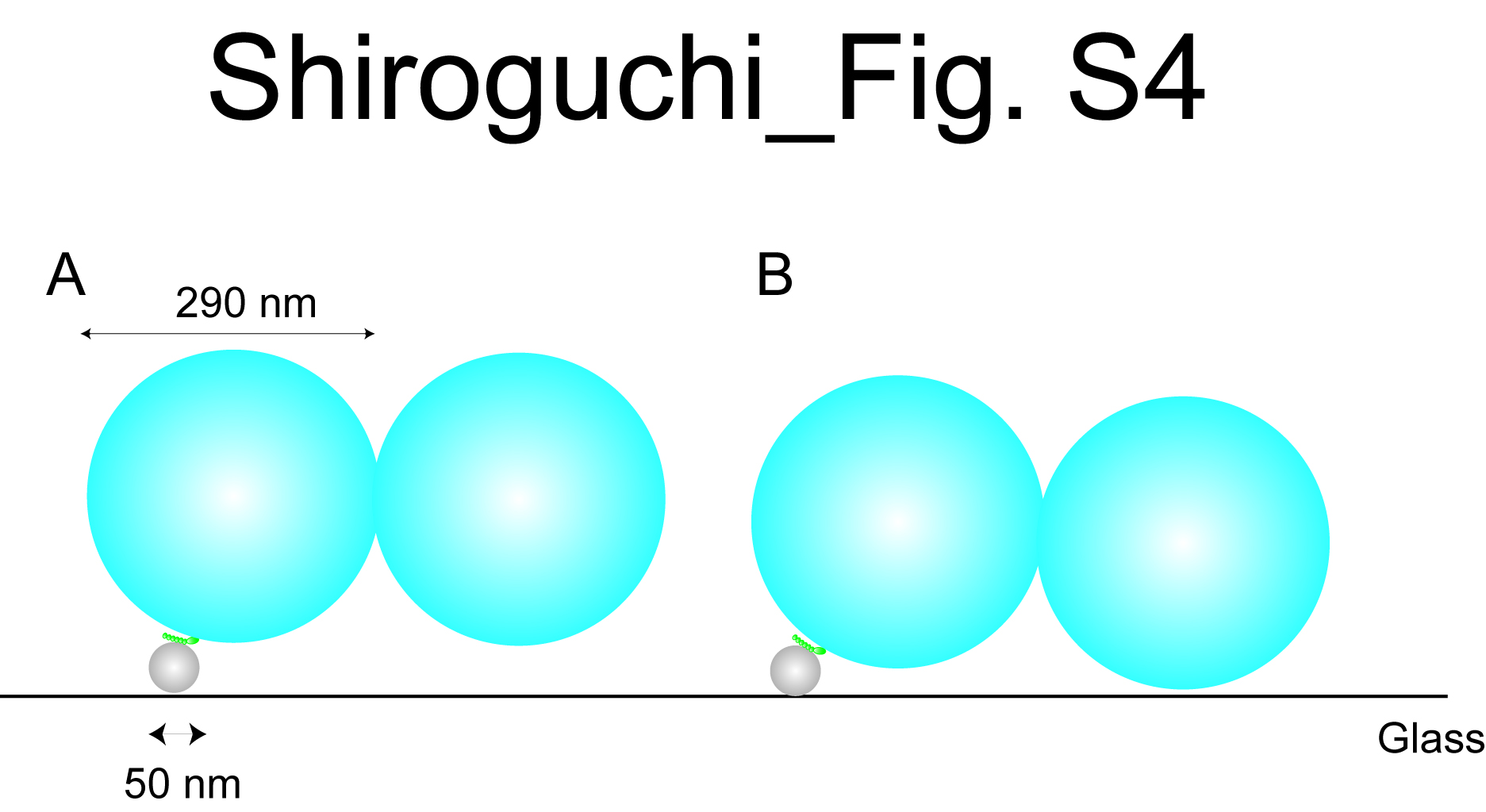

Supplement: Figure S4 — Configuration of myosin and beads drawn to scale. Examples of configurations in which large duplex beads can swing (A) and cannot swing (B). Myosin (in green) is between two sizes of beads: other proteins shown in Figure 1B are not shown here. The myosin neck is immobilized on a small gray bead, and the head is attached to a large blue duplex. Myosin binding to small and large beads occurs by chance. Duplex bead swinging occurs only when conditions under which the swinging beads do not collide with the surface are satisfied: (i) myosin is on the top of the small bead, (ii) myosin is properly oriented such that the swing plane is parallel to the surface, and (iii) the long axis of duplex beads is almost parallel to a surface. These conditions contribute to a low frequency of observed bead swinging. (JPG) [file pbio.1001031.s004.jpg]

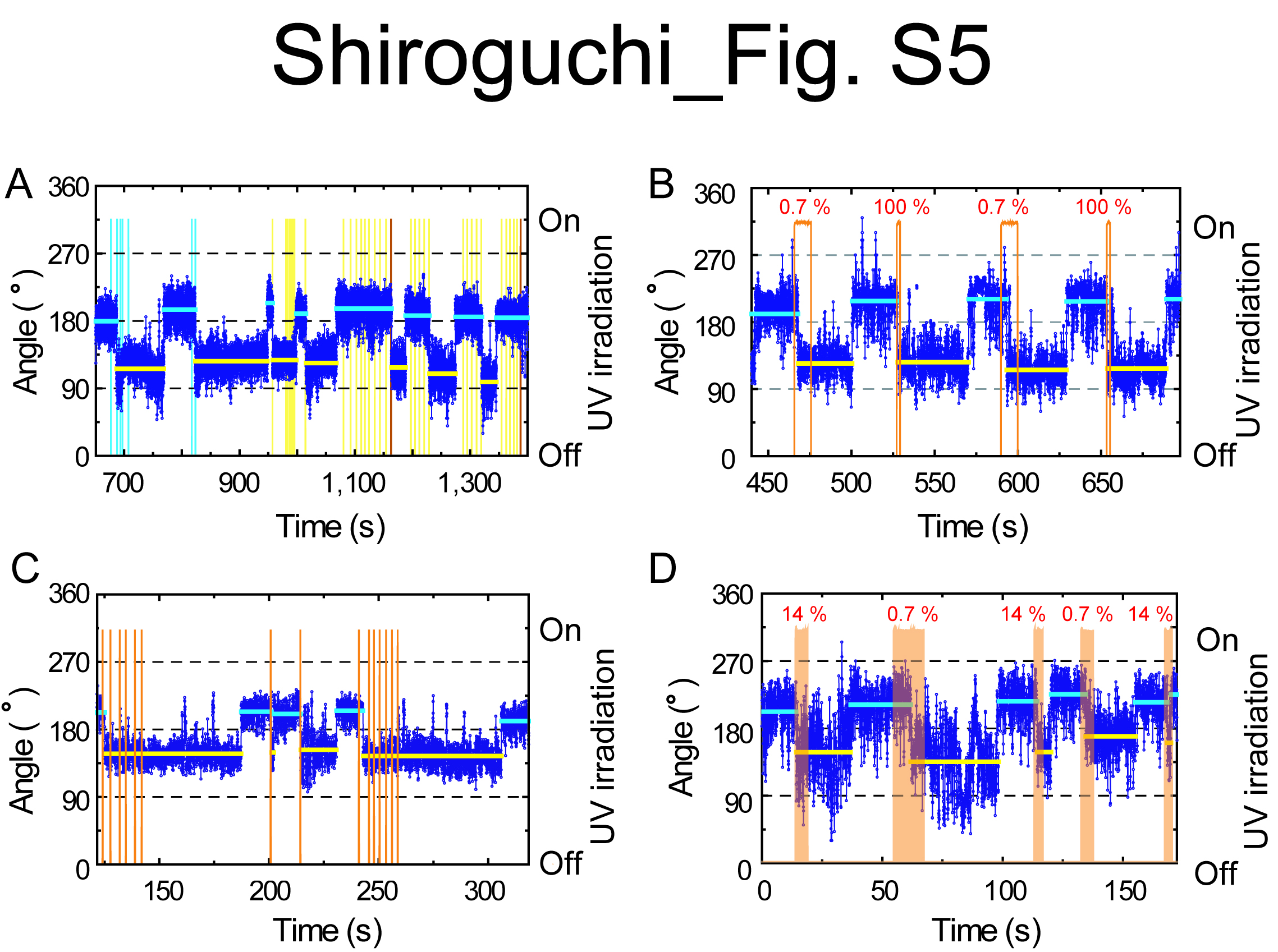

Supplement: Figure S5 — Head–neck swings of myosin Va under different UV irradiation conditions. Dark blue dots with a light gray line indicate angular positions of the beads on the head at 33-ms intervals (video frame rate); horizontal cyan lines indicate average angles before UV irradiation (pre-recovery stroke state); yellow lines indicate average angles after irradiation (post-recovery stroke state). (A) Single UV flashes of different powers. Vertical cyan lines indicate 100% intensity for 10 ms; yellow indicates 25% for 10 ms; brown indicates 25% for 100 ms. Compared to Figure 1C, where a 100-ms flash at 100% intensity always induced a return swing, short (10 ms) and/or weak (25%) flashes here often had to be applied several times before a successful return swing was observed, indicating that the swings depend on UV-generated ATP. (B) Continuous UV irradiation at 100% and 0.7% intensities for 2 s and 10 s, respectively. Under 100% UV, a swing was observed at 0.74 s on average (seven swings in three molecules), and under 0.7%, at 3.2 s (eight swings). Under continuous irradiation, [ATP] would rise toward the steady-state value of ∼50 µM at 100% (the generation rate of ∼20 µM divided by the depletion rate of 1/[2–3 s]) or ∼0.4 µM at 0.7%, with the time constant of 2–3 s (Figures S2 and S3). The observed waiting times above are thus consistent with ATP binding to myosin Va with the bimolecular rate constant of 1.7×10^6^ M−1s−1 measured in the stopped flow apparatus (Protocol S1). (C) UV flashes (100%, 0.2 s) in the post-recovery stroke state. None induced a swing back to the pre-recovery stroke state. (D) Quasi-steady ATP levels generated by the patterned irradiation in Figure 2A at indicated intensities. This is another example of the experiment in Figure 3A. (JPG) [file pbio.1001031.s005.jpg]

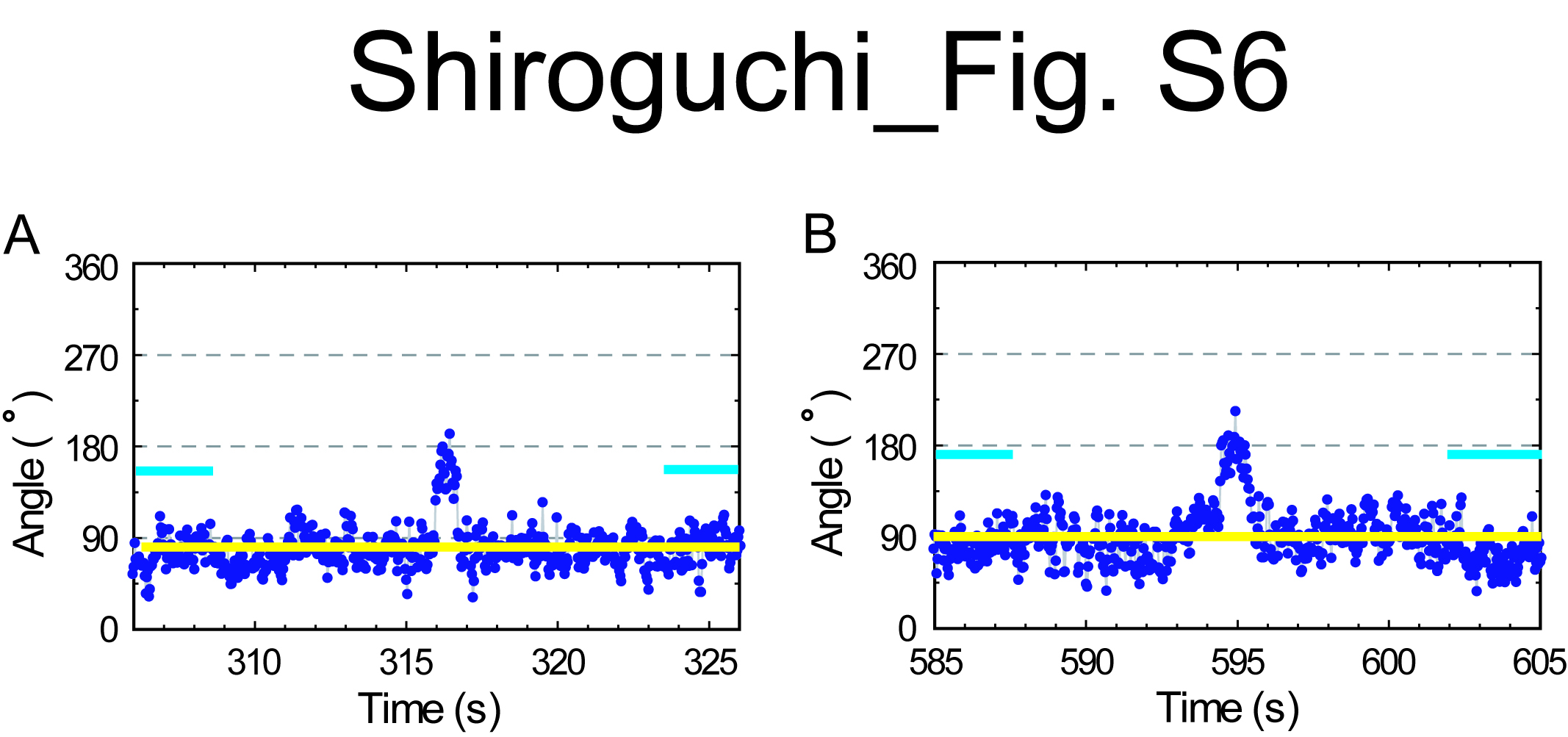

Supplement: Figure S6 — Momentary reversals to the pre-recovery stroke angle during post-recovery stroke states. (A and B) Dark blue dots with a light gray line indicate the angular positions of the beads on the head at 33-ms intervals (video frame rate); yellow indicates the average angle of the post-recovery stroke state; cyan indicates the pre-recovery stroke state before (left) and after (right) the shown post-recovery stroke state. These are expanded parts of the time course in Figure 1C, around two arrow heads. (JPG) [file pbio.1001031.s006.jpg]

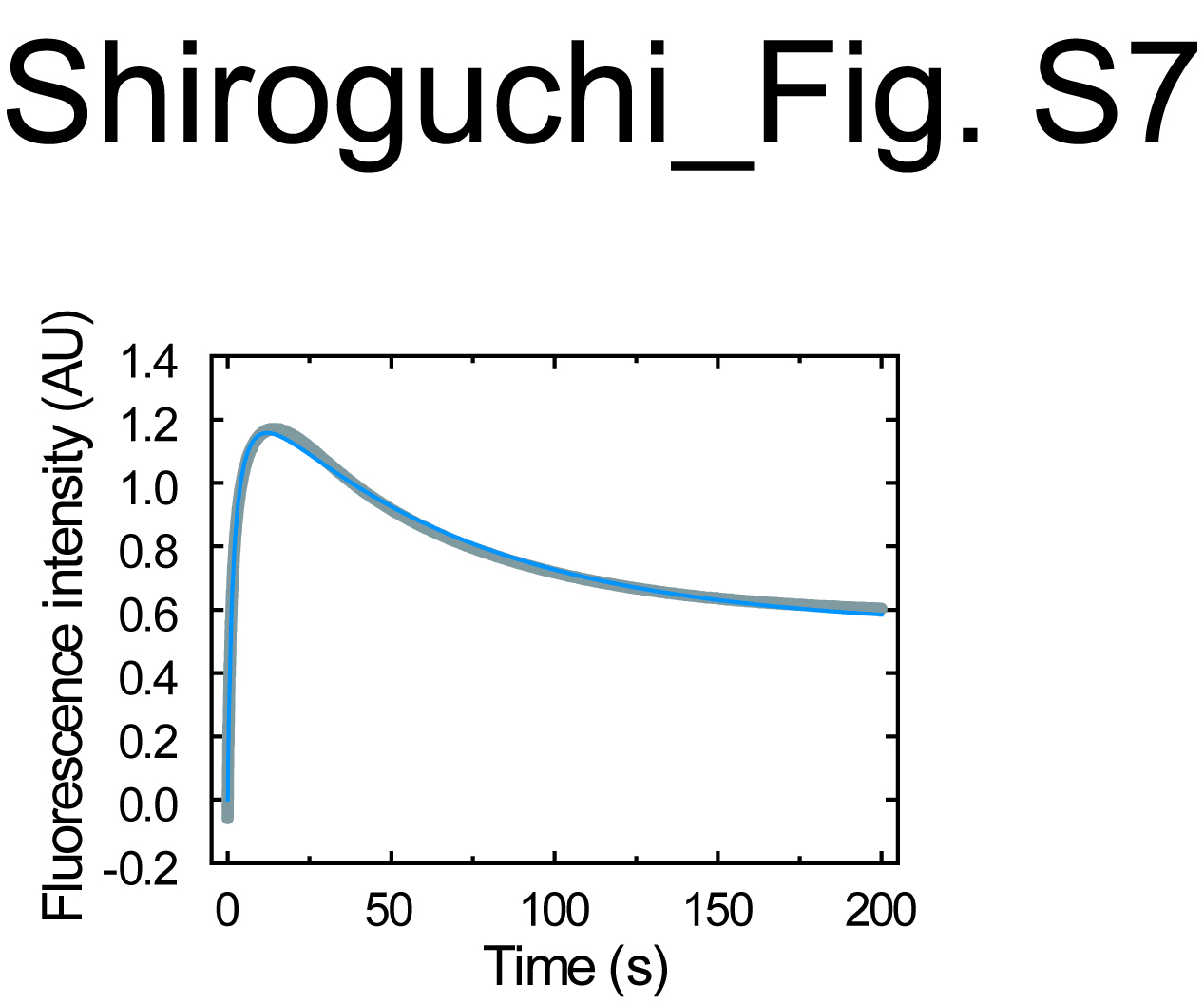

Supplement: Figure S7 — Time course of fluorescence change after mixing 1.0 µM monomeric myosin Va with 0.4 µM Mg-mantATP. The increase in fluorescence represents mantATP binding. The reduction results from mantADP release, which is limited by Pi release [23]. The data (gray) represent an individual, unaveraged time course of fluorescence change after subtraction of a baseline from mantATP photobleaching. The smooth line (cyan) through the data represents the best fit and yields a mantATP association rate constant of 1.57 (± 0.002) µM−1s−1 and a Pi release rate constant of 0.019 (± 0.001) s−1. A Pi release rate constant measured with ATP was 0.028 (± 0.001) s−1. These are consistent with our previous measurements for a shorter neck construct (Pi release, 0.02 s−1) [23]. (JPG) [file pbio.1001031.s007.jpg]

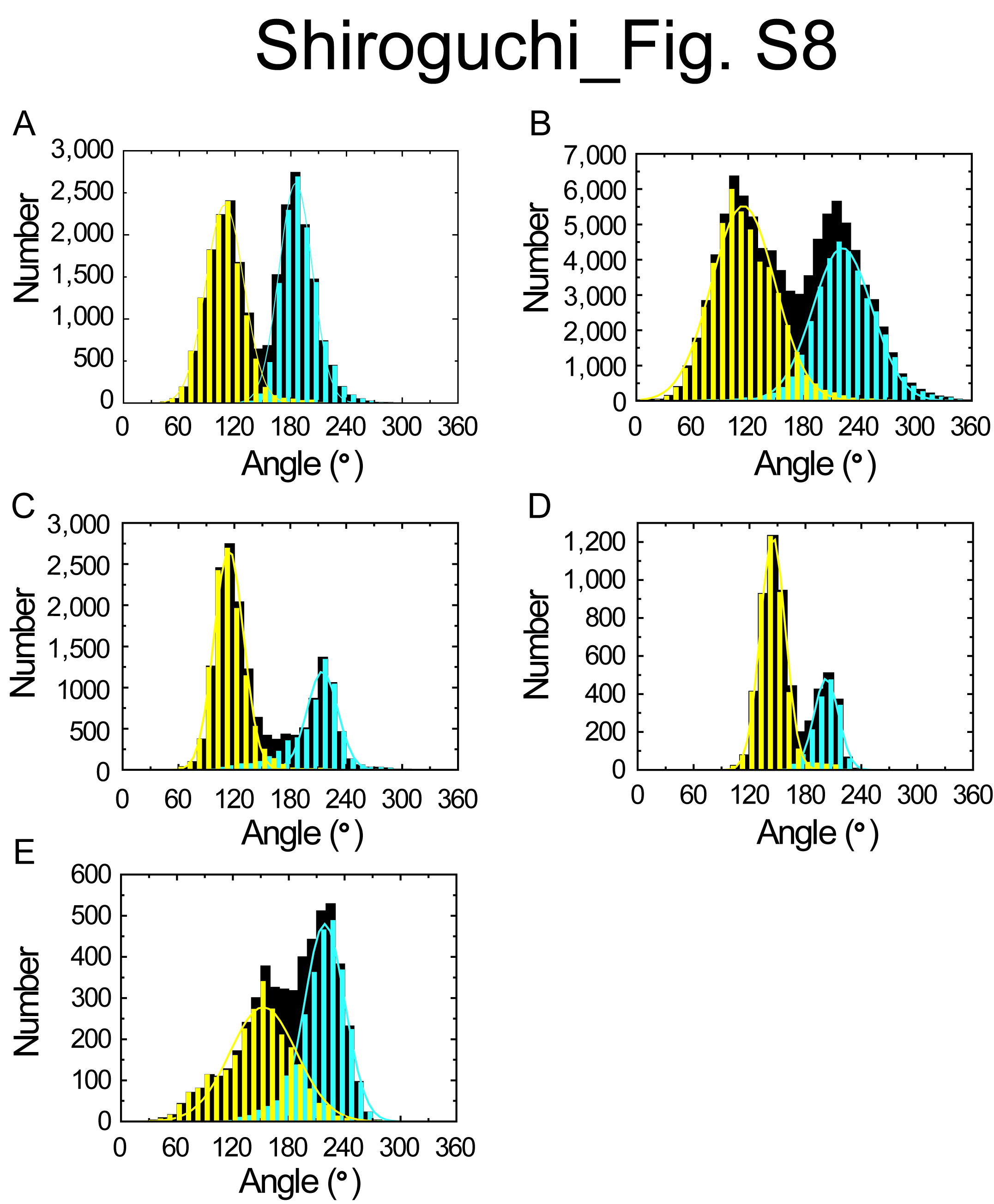

Supplement: Figure S8 — Distributions of bead angles in the pre-recovery stroke (cyan) and post-recovery stroke (yellow) states. Black bars indicate whole frames. These are additional examples of the analysis in Figure 5A. (A–E) Distributions for Figures 1C, 3A, and S5B–S5D, respectively. Lines show Gaussian fits: exp[−(θ − θm)2/2σ2] where θm is the mean angle. (JPG) [file pbio.1001031.s008.jpg]
