# Supplementary material for: Direct Observation of the Myosin Va Recovery Stroke That Contributes to Unidirectional Stepping along Actin
Source: PLoS Biol. 2011 Apr 12;9(4):e1001031. doi: 10.1371/journal.pbio.1001031 (PMC3075224; doi:10.1371/journal.pbio.1001031)
Supplement: Protocol S1 — Materials, gliding bead assay, F1-ATPase rotation assay, and transient kinetic analysis. The detailed protocols are described. (DOC) [file pbio.1001031.s009.doc]

**Protocol S1**

**Materials.** Native myosin V was purified from chick brain as previously reported (1). Rabbit skeletal actin was prepared (2), biotinylated (biotin-(AC_5_)_2_-Sulfo-Osu, Dojindo Laboratories), and stained with Alexa Fluor 488 phalloidin (ref. 3) (Invitrogen). Biotinylated α-casein was made by mixing unphosphorylated α-casein (Sigma) with biotin-(AC_5_)_2_-Sulfo-Osu and was purified by gel chromatography. A GT mutant (4, 5) of the rotary motor F_1_-ATPase was expressed in *E. coli* and its rotor (the γ subunit) was biotinylated at the two genetically introduced cysteines.

**Gliding bead assay.** The gliding velocity of myosin-coated beads on surface-immobilized actin filaments was examined to estimate the concentration of ATP generated by UV irradiation. All solutions below were in buffer A and the following infusions (2-3 chamber volumes) were made with 1-2 min incubation times in between: 0.13 mg mL^-1^ biotinylated unphosphorylated α-casein, 2 mg mL^-1^ unbiotinylated, unphosphorylated α-casein, 1 mg mL^-1^ streptavidin, buffer A for washing, 200 nM biotinylated alexa 488 labeled actin, buffer A for washing. Finally, native myosin V-bead complex (myosin V mixed with 0.34 μm carboxylated polystyrene beads (Polysciences) at the molar ratio of 5:1 and incubated for more than 2 min) was infused together with 200 μM caged ATP, 1.7 mU μL^-1^ apyrase, 1 mg mL^-1^ unphosphorylated α-casein, an oxygen scavenger system (3.2 mg mL^-1^ glucose, 0.2 mg mL^-1^ glucose oxidase, and 0.89 mg mL^-1^ catalase), and 0.5 % (v/v) β-mercaptoethanol. UV irradiation was initiated after the myosin-coated beads had settled on the surface-immobilized actin filaments.

**F_1_–ATPase rotation assay.** The GT mutant of F_1_-ATPase which, unlike the wild type, does not readily adopt an inhibited state (4, 5) was adsorbed on a clean glass surface and the rotation of a streptavidin-coated bead duplex attached to the biotinylated rotor (γ subunit) was observed (Figure S3A) (ref. 6). All solutions below were in buffer A and the following infusions (2-3 chamber volumes) were made with 1-2 min incubation in between: 4 -20 nM GT mutant, 5 mg mL^-1^ BSA, 0.29-μm streptavidin coated beads (Seradyn, washed three times by centrifugation with buffer A) together with 200 μM ATP (Sigma) and 3.8 mg mL^-1^ BSA, buffer A for washing. Finally, 200 μM caged ATP and 1.7 mU μL^-1^ apyrase. Rotating beads were identified upon UV excitation. After allowing apyrase to deplete generated ATP, we monitored rotation under several UV irradiation conditions (Figures 2, S3). In the case of ATP-dependent rotation, bead solution did ont contain ATP, and the final infusion solution contained ATP (0.05 – 0.5 μM) and an ATP-regenerating system (1 mM creatine phosphate and 0.1 mg mL^-1^ creatine kinase) instead of caged ATP and apyrase.

**Transient kinetic analysis.** Transient kinetic fluorescence measurements were performed with single-headed myosin Va (7) using an Applied Photophysics SX.18MV-R stopped flow apparatus thermostatted at 25±0.1 ºC. All experiments were performed in KMg50 buffer (50 mM KCl, 2 mM MgCl_2_, 1 mM EGTA, 1 mM DTT, and 10 mM imidazole, pH 7.0). A molar equivalent of MgCl_2_ was added to ATP and mant-ATP immediately before use. Uncertainties are reported as standard errors in the fits. Concentrations stated are final after mixing.

Mant-ATP binding kinetics were measured by Förster resonance energy transfer from myosin V tryptophan residues (λ_ex_=280 nm) to bound fluorescent mant-nucleotide under pseudo-first-order conditions with [nucleotide] >> [myosin V] (ref 7, 8). Fluorescence was monitored at 90° through a 400-nm, long-pass colored glass filter. The assay was performed twice or more in each ATP concentration (5, 10, 15, 20, 25, 35, 50 and 125 μM). Time courses of nucleotide binding were fit to single exponentials using software provided with the instrument. The ATP association rate constant of 1.7 (± 0.1) ×10^6^ M^-1^s^-1^ was determined from the best linear fit of the concentration-dependence of the observed reaction rate constants (9).

Single turnover experiments were performed by mixing 0.4 μM ATP or mant-ATP (λ_ex_ = 280 nm) with excess (1 μM) monomeric myosin V. Fluorescence was monitored at 90° through a Schott 320WG filter or a 400-nm, long-pass colored glass filter for ATP or mant-ATP binding, respectively. Time courses were fit by numerical simulation using KinTek Global Kinetic Explorer following a three-step model with initial ATP binding under non-pseudo-first order conditions followed by slow, rate-limiting P*_i_* release then rapid ADP release (7). The ATP hydrolysis rate constant is rapid (7) and does not contribute to the observed relaxations.

**References for Protocol S1**

1. Cheney RE (1998) Purification and assay of myosin V. *Methods Enzymol.* **298:** 3-18.
2. Spudich JA, Watt S (1971) The regulation of rabbit skeletal muscle contraction. I. Biochemical studies of the interaction of the tropomyosin-troponin complex with actin and the proteolytic fragments of myosin. *J. Biol. Chem.* **246:** 4866-4871.
3. Ali MY, *et al.* (2002) Myosin V is a left-handed spiral motor on the right-handed actin helix. *Nat. Struct. Biol.* **9:** 464-467.
4. Nishizaka T, *et al.* (2004) Chemomechanical coupling in F_1_-ATPase revealed by simultaneous observation of nucleotide kinetics and rotation. *Nat. Struct. Mol. Biol.* **11:** 142-148.
5. Muneyuki E, *et al.* (2007) Single molecule energetics of F_1_-ATPase motor. *Biophys. J.* **92:** 1806-1812.
6. Yasuda R, Noji H, Yoshida M, Kinosita K Jr, Itoh H (2001) Resolution of distinct rotational substeps by submillisecond kinetic analysis of F_1_-ATPase. *Nature* **410:** 898-904.
7. De La Cruz EM, Wells AL, Rosenfeld SS, Ostap EM, Sweeney HL (1999) The kinetic mechanism of myosin V. *Proc. Natl. Acad. Sci. USA* **96:** 13726-13731.
8. Henn A, De La Cruz EM (2005) Vertebrate myosin VIIb is a high duty ratio motor adapted for generating and maintaining tension. *J. Biol. Chem.* **280:** 39665-39676.
9. De La Cruz EM, Ostap EM. (2009) Kinetic and equilibrium analysis of the myosin ATPase. *Methods Enzymol*. **455** 157-92.
